# Evolution of Quality of Life and Treatment Adherence after One Year of Intermittent Bladder Catheterisation in Functional Urology Unit Patients

**DOI:** 10.3390/jcm12082928

**Published:** 2023-04-18

**Authors:** Blanca Fernandez-Lasquetty Blanc, Antonio Hernández Martínez, Carlos Lorenzo García, Montserrat Baixauli Puig, Francisco Estudillo González, Maria Victoria Martin Bermejo, Maria Angustias Ortega Checa, Elena Alcaraz Zomeño, Arancha Torres Bacete, Guillermina Ferrández Franco, Begoña Benito Santos, Guadalupe Fernández Llorente, Maria Carmen Guerrero Andrádes, Monica Rodríguez Diaz, Mario Pierre Louis Lauture, Isabel Jiménez Mayorga, Rosario Serrano-Abiétar, Maria Asunción Garrido Mora, Francisco Barcia Barrera, Gemma Asensio Malo, Montserrat Morcillo Marín, Vicenta Lluesma Martinez, Maria Luisa Valero Escribá, Silvia Tendero Ruiz, Rosa Ana Romay Cea, Mercedes Marín Valero, Julián Rodríguez-Almagro

**Affiliations:** 1 Nursing and Nutrition Department, Universidad Europea de Madrid, 28670 Madrid, Spain; 2 Department of Nursing, Physiotherapy and Occupational Therapy, Ciudad Real Faculty of Nursing, University of Castilla-La Mancha, 13071 Ciudad Real, Spain; 3 Department of Nursing, Hospital Universitario Clínico San Carlos, 28040 Madrid, Spain; 4 Department of Nursing, Hospital Universitari Clinic Barcelona, 08036 Barcelona, Spain; 5 Department of Nursing, Hospital Universitario Puerto Real, 11510 Cadiz, Spain; 6 Department of Nursing, Hospital Nacional de Paraplejicos, 45004 Toledo, Spain; 7 Department of Nursing, Hospital Universitari I Politecnic La Fe, 46026 Valencia, Spain; 8 Department of Nursing, Hospital Universitario Ramón y Cajal, 28034 Madrid, Spain; 9 Department of Nursing, Hospital Universitario Infanta Leonor, 28031 Madrid, Spain; 10 Hospital General Universitario de Alicante, 03010 Alicante, Spain; 11 Department of Nursing, Hospital Universitario Infanta Sofía, 28702 Madrid, Spain; 12 Department of Nursing, Hospital Universitario Puerta del Mar, 11009 Cadiz, Spain; 13 Department of Nursing, Hospital Universitario Virgen de las Nieves, 18014 Granada, Spain; 14 Department of Nursing, Hospital Universitario Virgen Macarena, 41009 Sevilla, Spain; 15 Department of Nursing, Hospital Universitario Regional de Malaga, 29010 Malaga, Spain; 16 Department of Nursing, Hospital Universitario Puerta de Hierro Majadahonda, 28222 Madrid, Spain; 17 Department of Nursing, Hospital General Universitario de Elche, 03203 Valencia, Spain; 18 Department of Nursing, Hospital Universitario Virgen del Rocio, 41013 Sevilla, Spain; 19 Department of Nursing, Hospital Universitari de Bellvitge, 08907 Barcelona, Spain; 20 Department of Nursing, Institut Guttmann, 08916 Barcelona, Spain; 21 Department of Nursing, Hospital Universitario de Fuenlabrada, 28942 Madrid, Spain; 22 Department of Nursing, EOXI a Coruña, 15009 A Coruña, Spain

**Keywords:** intermittent catheterisation, adherence, self-care, quality of life

## Abstract

Objective: To determine patient difficulties and concerns when performing IBC (Intermittent Bladder Catheterisation), as well as the evolution of adherence, quality of life, and emotional state of patients one year after starting IBC. Method: A prospective, observational, multicentre study conducted in 20 Spanish hospitals with a one-year follow-up. Data sources were patient records and the King’s Health Questionnaire on quality of life, the Mini-Mental State Examination (MMSE), and the Hospital Anxiety and Depression Scale (HADS). Perceived adherence was measured using the ICAS (Intermittent Catheterization Adherence Scale) and perceived difficulties with IBC were assessed using the ICDQ (Intermittent Catheterization Difficulty Questionnaire). For data analysis, descriptive and bivariate statistics were performed for paired data at three points in time (T1: one month, T2: three months, T3: one year). Results: A total of 134 subjects initially participated in the study (T0), becoming 104 subjects at T1, 91 at T2, and 88 at T3, with a mean age of 39 years (standard deviation = 22.16 years). Actual IBC adherence ranged from 84.8% at T1 to 84.1% at T3. After one year of follow-up, a statistically significant improvement in quality of life (*p* ≤ 0.05) was observed in all dimensions with the exception of personal relationships. However, there were no changes in the levels of anxiety (*p* = 0.190) or depression (*p* = 0.682) at T3 compared to T0. Conclusions: Patients requiring IBC exhibit good treatment adherence, with a significant proportion of them performing self-catheterisation. After one year of IBC, a significant improvement in quality of life was noted, albeit with a significant impact on their daily lives and their personal and social relationships. Patient support programmes could be implemented to improve their ability to cope with difficulties and thus enhance both their quality of life and the maintenance of their adherence.

## 1. Introduction

Intermittent bladder catheterisation (IBC) involves periodically emptying the urinary bladder by inserting a catheter through the urethra and removing it when voiding is complete. This procedure may be indicated in both the short and long term for conditions such as urinary retention or neurogenic bladder dysfunction (e.g., spinal cord injury, myelomeningocele, atonic bladder) [1,2,3]. IBC is therefore considered the treatment of choice for voiding dysfunction with chronic urinary retention and to protect the upper urinary tract [4,5]. It also provides greater independence for the individual and improves aspects such as social, work, and school integration, couple relationships, self-esteem, and overall quality of life [6,7,8]. This procedure therefore not only promotes patient autonomy, but also helps to preserve renal function and maintain the patient’s urological health.

However, a number of authors have identified different factors that may play a role in the lack of IBC adherence. These include lack of privacy and suitable spaces in public places, as well as the need for assistance to perform the procedure, lack of time, and the need for catheterisation planning [8,9,10,11]. Additionally, another phenomenon observed is that certain patients do not follow the recommendations for IBC, especially with regard to the number of catheterisations prescribed and long-term compliance [11].

Furthermore, although the relationship between IBC and the improvement of patient quality of life and emotional state has been previously explored, few studies have been published on the topic [12,13,14], most of them using qualitative designs [13,15,16,17,18] and small sample sizes. In 2021, our research team published the results of a pilot study where these aspects were evaluated after a one-month follow-up of IBC, yielding positive results; however, we ignored whether these results can be maintained in the long term [14].

For these reasons, the aim of this study was to identify the difficulties and concerns surrounding the implementation of IBC, as well as the evolution of patient adherence, quality of life, and emotional state one year after initiating IBC.

## 2. Method

### 2.1. Design

This is a prospective, observational, multicentre study that was carried out in 20 hospitals across 11 Spanish cities from 15 October 2020 to 15 December 2021. The study started with a cohort of patients whose results were published in 2021 [14].

### 2.2. Selection of Study Subjects

The reference population consisted of patients who performed IBC and were seen at the functional urology units participating in the study. The inclusion criteria were being 18 years of age or older and that this was the first time that catheterisation was prescribed to them. The exclusion criteria included language barriers and cognitive and/or sensory impairments preventing patients from performing IBC and understanding the purpose of the study.

As a result, the study population consisted of patients from the reference population who met the inclusion criteria and voluntarily agreed to participate. The criteria for patient withdrawal from the study were the following: a change of hospital, a decision to withdraw from the study, end of treatment, or exitus during the study.

### 2.3. Sample Size Calculation

The Granmo tool was used to estimate the required sample size (https://www.imim.es/ofertadeserveis/software-public/granmo/, accessed on 1 February 2023). We took the following into consideration: a 95% confidence level; an unknown reference population susceptible to requiring IBC because of their particular health conditions; a prevalence of 50%, which is the most demanding for estimating a sample size; a 10% precision error; and a 10% replacement rate. These criteria resulted in a sample size of 107 study subjects.

### 2.4. Sources of Information and Study Variables

Patient records and a data collection notebook developed specifically for this study were used as sources of information. This notebook included a questionnaire of our own design that assessed the frequency and level of difficulties performing IBC, as well as validated tools to assess the following: cognitive state, using the Mini-Mental State Examination [19] (where lower scores indicate poorer cognitive states); anxiety and depression, using the Hospital Anxiety and Depression Scale or HADS [20] (where higher scores indicate higher levels of anxiety and depression); quality of life linked to urinary problems, using King’s Health Questionnaire [21] (where higher scores indicate a poorer quality of life); perceived adherence, using the ICAS (Intermittent Catheterization Adherence Scale) [22]; and perceived difficulties in performing IBC, using the modified ICDQ (Intermittent Catheterization Difficulty Questionnaire) [23].

The main outcome variable of the study was treatment adherence, understood as the maintenance of the number of IBCs prescribed by the practitioner. Other outcome variables were perceived IBC adherence as measured using the ICAS; changes in quality of life according to King’s Health Questionnaire; and changes in emotional state based on the HADS. The independent variables were sociodemographic, anthropometric, and clinical in nature, as well as determinants of previous cognitive state, previous anxiety, previous depression, and previous quality of life.

### 2.5. Patient Recruitment and Follow-Up

Patients who had just received a prescription for IBC were contacted by the nurse researchers from the participating facilities. They invited all patients to participate in the study and informed them of all the study details following a non-probabilistic consecutive sampling method.

Once they agreed to participate and signed the informed consent form, participants were asked for additional data that are not usually included in clinical records.

The participating nurses carried out their care work by briefing and training patients to comply with IBC treatment following the standard guidelines of each unit/department. At that time, they filled in the data collection notebook together with the HADS, the Mini-Mental Status Examination, and King’s Health Questionnaire (T0).

The nurses then phoned the patients for a one-month follow-up (T1), for a 3-month follow-up (T2), and for a 12-month follow-up (T3) to ascertain whether they continued performing IBC and their reasons for discontinuation, if applicable. In each of these telephone calls, the following were completed: the follow-up notebook, the quality-of-life questionnaire (King’s Health Questionnaire), the anxiety and depression questionnaire (HADS), the perceived adherence scale (ICAS), and the questionnaire of perceived difficulties in performing IBC (modified ICDQ).

### 2.6. Statistical Analysis

Firstly, descriptive statistics were performed for all the study variables: absolute and relative frequencies for qualitative variables; and means and standard deviations for quantitative variables with a normal distribution, or alternatively, medians and interquartile ranges.

Changes in perceived difficulties performing IBC, the evolution of adherence, and ICAS adherence scores between the first month and one year after the initial IBC were then assessed using Wilcoxson’s non-parametric test for paired data, McNemar’s test, or Student–Fisher’s *t*-test for paired data, depending on whether the variable in question was ordinal, dichotomous, or quantitative in nature. Lastly, changes in quality of life and changes in anxiety/depression levels between baseline (T0) and 12 months (T3) were assessed using Student–Fisher’s *t*-test for paired data. The SPSS 28.0 statistical package was used for all analyses.

### 2.7. Ethical and Legal Considerations

This observational study was based on anonymised data and designed in accordance with the Declaration of Helsinki as laid out by the World Medical Association (WMA). The study was approved by the Clinical Research Ethics Committees at the recruitment hospitals.

Patients were invited to participate in the study and received a patient information sheet. The particulars of the study were verbally explained to them, and they were required to sign the informed consent form. If patients wished to take part in the study, their personal data were attributed numerical codes for confidentiality.

Additionally, the study complied with the Spanish Organic Law 3/2018 of 5 December, on Personal Data Protection and Guarantee of Digital Rights, ensuring the anonymity of the participants and the database, with no personally identifiable data.

## 3. Results

### Characteristics of the Subjects Included in the Study

A total of 134 subjects initially participated in the study (T0), becoming 104 subjects at T1, 91 subjects at T2, and 88 subjects at T3. The most relevant sociodemographic characteristics of the patients at the beginning of the study included a mean age of 39.0 years (SD = 22.16 years), with 55.2% (n = 74) of the sample being male, 45.5% (n = 61) having a primary education, and 46.3% (n = 62) being retired; 88.1% (118) were living at home with family and/or caregiver support. Table 1 provides all of the patient sociodemographic information.

In terms of clinical characteristics, it was observed that 32.8% (n = 44) of participants presented neurological damage for which IBC was prescribed, followed by impaired contractile function, at 30.6% (n = 41). Cardiovascular conditions ranked first, at 24.6% (n = 33) of pre-existing conditions, followed by musculoskeletal conditions, at 19.4% (n = 26). Comorbidities included obesity, at 14.2% (19), followed by previous depression and anxiety, both at 9.7% (n = 13). Catheterisation was prescribed by a urologist in 91.0% (n = 122) of patient cases and by a nurse in 11.2% (n = 15) of them. The clinical characteristics can be found in Table 2.

Regarding their ability to self-care, hand function was assessed, revealing that only 5.2% (n = 7) had limited motor skills in both hands, 2.2% (n = 3) in the dominant hand only, and 11.2% (n = 15) had limited sensation in both hands. Mobility was also assessed and only 60.4% (n = 81) were found to have normal mobility. Additionally, 20.9% (n = 28) of patients reported difficulty in locating their urinary meatus. Regarding their ability to repeat and understand the information provided on IBC, most patients (97.0%; n = 130 and 92.5%; n = 124, respectively) were found to be able to do so. In this first assessment, 88.1% (n = 118) of patients believed that they would need help to perform IBCs (Table 3).

Patients were then surveyed about their concerns regarding the use of IBC. A percentage assessment was made based on their degree of concern on a Likert scale ranging from ‘No concern’ to ‘Very concerned’. Focusing on the ‘Very concerned’ category, the most concerning aspect for patients was the risk of infection (5.2%; n = 7), with this item also having the highest mean and median scores when considering all the response options together. As for the category ‘No concern’, the aspect with the highest percentage of responses was the feeling of losing masculinity or femininity, with 71.6% (n = 96), which also coincides with the lowest mean and median scores. Table 4 provides this information in further detail.

Regarding cognitive state, 91.0% (n = 122) obtained normal values, and there was only 1 subject with scores consistent with dementia. In terms of quality of life, the most affected dimensions were incontinence impact and personal relationships, and 11.9% (n = 16) cases of anxiety and 8.2% (n = 11) cases of depression were observed. These, and the rest of the related data can be consulted in Table 5.

After one month of follow-up, the researchers contacted the patients to assess their condition, to ascertain whether they continued performing IBC, and to discuss their experience and how this had impacted their quality of life and their levels of anxiety and depression (Table 6).

Firstly, they were asked about the type of catheter they had used, with 72.4% (n = 79) using a ready-to-use hydrophilic catheter. Interestingly, 79.8% (n = 95) of patients performed their IBC independently, despite the fact that, at the beginning of the study, most of them believed that they would need assistance.

The difficulties patients had experienced in performing bladder catheterisation, both in terms of level and frequency were also analysed. Although patients were originally asked a 4-point Likert question (ranging from 0 to 3 points), the mean score for each suggested difficulty was calculated to create an overall picture of the most problematic aspects. Specifically, the aspect with the highest mean score was ‘Public bathrooms do not meet hygienic requirements’, followed by ‘I could not find a private place’ both in terms of frequency and level. The evolution of the average difficulty scores both in terms of frequency and level during the follow-up period is presented below. Regarding the changes experienced, there was only a statistically significant improvement between T1 and T3 in ‘locating the urinary meatus’ and ‘catheterisation using the no-touch technique’. In contrast, there was a statistically significant worsening of ‘problems accessing public toilets’ and ‘not finding a private place’. All information about difficulties experienced with IBC can be found in Table 7.

Actual IBC adherence (maintaining the prescribed number of catheterisations) and adherence—as assessed using the quantitative and categorical versions of the ICAS—did not experience statistically significant changes (*p* > 0.05) throughout the follow-up period between T1 and T3. As a result, actual adherence values ranged from 84.8% at T1 to 84.1% at T3.

Finally, changes in quality of life and in the anxiety/depression scale during the one-year follow-up were assessed by comparing the baseline situation (recruitment visit, T0) with the last visit (T3; n = 79). As shown in Table 8, there was a statistically significant improvement in quality of life (*p* ≤ 0.05) (lower King’s Health scores) in all dimensions with the exception of personal relationships. However, there were no changes in levels of anxiety (*p* = 0.190) or depression (*p* = 0.682) at visit T3 compared to T0 (Table 9).

## 4. Discussion

The main findings of our study include, firstly, the great variability of clinical and sociodemographic characteristics of IBC users, which is in line with other studies [24,25]. As such, although most of our sample exhibited a good mental state and were able to follow the instructions for proper IBC, more than half of the patients had some type of mobility issue and a number of them even had difficulty locating the urinary meatus. However, despite this, almost 80% performed self-catheterisations, which is in line with what other authors have reported [6,26]. In fact, IBC is a procedure that can be performed by individuals of all ages, including the elderly and children from 4–5 years of age under adult supervision. Training and close monitoring by nurses specialising in functional urology is essential to performing IBC properly. These nurses not only train patients in the performance of the technique, but also in their self-care and in the successful integration of the treatment into their daily lives. One of the results of our study is a reduction in the difficulty in locating the urinary meatus and in the ‘no touch’ technique throughout the follow-up period. This is supported by a recent patient support study that showed an increase in IBC adherence as well as a decrease in the number of related emergency consultations and hospitalisations [27]. Patient support programmes are of great interest, especially at the beginning of treatment, when patients tend to drop out due to difficulties performing the technique or because of fear of IBC [16]. Such training should have a strong health education component, aiming to dispel concerns about IBC, such as the fear of infection, which is the main concern reported by the patients in our study. Training is therefore considered a key determinant of treatment adherence [28].

Furthermore, the participants in this study reported a great variability in the equipment used for IBC, employing up to 7 types of catheters made of different materials and with different technical features. The reason for choosing one type of catheter over another is to promote IBC adherence and avoid related complications, always depending on the preferences and clinical characteristics of each patient. We therefore concur with other authors that the choice of equipment should be tailored to the needs and preferences of the patient and comfort criteria for catheter handling according to the model [28]. Restricting the type of equipment used could result in treatment abandonment, especially in the early stages, as well as complications such as haematuria or urethrorrhagia. For instance, when a patient is at risk of urethral microtrauma, it is recommended that the patient be trained in the use of hydrophilic catheters [29].

Another of the aspects explored in this study were the difficulties and/or barriers reported by the patients, who highlighted that public toilets did not meet the required hygienic conditions for catheterisation and that they could not find a private place to conduct catheterisation in public spaces. The same barriers were identified by Cobussen et al. [15]. These problems illustrate the need to select equipment for patients that is as sterile and easy to use as possible, facilitating a ‘no touch’ technique, while avoiding disruption of their social life (travel, leisure activities, work), and thereby improving their quality of life.

Despite the variability in both the profile of patients performing IBC and the difficulties observed, there was a high percentage of IBC adherence (84.1% at one-year follow-up). This percentage is higher than those published by other authors such as Hentzen et al. [30] (66.9%) and Montavaselli et al. [31] (29%) at one-month follow-up, as well as a study by Girotti et al. [32] (58%).

We therefore believe that this success is a consequence of the strong involvement of specialised nurses in patient training. This conclusion is supported by other studies such as the one published by Hasan et al. [27] in 2022, where, after implementing a patient support programme, adherence improved (up to 88% at one-month follow-up), and the number of visits to the emergency department also decreased in the first month of follow-up.

Regarding the impact of IBC on quality of life and mood, we observed a significant short-term improvement in quality of life, in agreement with other authors [13]. However, these authors also acknowledged that it is crucial for patients, especially older people, to receive adequate support from trained nurses in the early stages. Our study shows that, although IBC treatment generally improves the quality of life of individuals, it has a significant impact on their daily lives and their personal and social relationships. This would justify the implementation of support programmes to help patients cope better with their problems and thus improve both their quality of life and the maintenance of adherence. However, there appears to be clear evidence that intermittent self-catheterisation is the technique that provides the most positive outcomes in terms of quality of life, as reported in the 2022 systematic review by Gharbi et al. [33] involving 25 studies and 3002 patients with neurogenic bladder dysfunction.

There was also an improvement in the initially published cut-off levels of anxiety/depression [14]. However, we observed no differences at one-year follow-up. Training may have a positive effect in the early stages, but it may stabilise or even deteriorate over time.

For this reason, it would be interesting to work on all these aspects in patient support programmes as suggested by other authors [17], since higher levels of anxiety/depression, a greater impact on the patient’s normal daily life, and disruption of personal relationships have been linked to poorer IBC adherence [31].

One of the main limitations of our study was the loss of 20 subjects as a consequence of the COVID-19 pandemic. However, despite these limitations, our study recruited more subjects than most published studies [13,15,16,17,18,32], most of them being qualitative and short-term in nature [13,15,16,17,18]. Finally, we believe that the results obtained may vary depending on the population selected. For instance, if there are many patients with poor motor skills who require assisted catheterisation, they may not perform as well as if catheterisation was performed on their own, as noted in a review by Gharbi et al. [33].

One of the main strengths of this study is that it addresses a topic with scarce international studies and is one of the few to cover a one-year follow-up period. Moreover, this is the first study to examine this problem in Spain taking a multicentre approach, in which patients from 20 Spanish hospitals are represented. Subject selection was rigorous and systematic, which is why we believe that any confounding bias may be minimal. In addition, validated and widely used tools were used to measure the phenomena under study.

## 5. Conclusions

In light of the above, we believe that patients requiring IBC exhibit good treatment adherence, with a significant proportion of them performing self-catheterisation. After one year of IBC, a significant improvement in quality of life was noted, albeit with a significant impact on their daily lives and their personal and social relationships Nevertheless, it appears necessary to offer patients support programmes that also address emotional issues and coping skills to improve their quality of life and the maintenance of their adherence.

## Figures and Tables

**Table 1:** Sociodemographic characteristics of the participating patients.

Variable	Initial Cohort T0 n (%) n = 134
Age in years (mean ± SD)	39.0 (22.16)
Sex	
Male	74 (55.2)
Female	60 (44.8)
Level of education	
No education	5 (3.7)
Primary education	61 (45.5)
Secondary education	35 (26.1)
University education	33 (24.6)
Occupation	
Retired	62 (46.3)
On leave	27 (20.1)
Leave of absence	2 (1.5)
Unemployed	10 (7.5)
Employed	33 (24.6)
Marital status	
Married	90 (67.2)
Divorced	4 (3.0)
Separated	3 (2.2)
Single	30 (22.4)
Widow(er)	7 (5.2)
Living situation	
Lives at home alone	13 (9.7)
Lives at home with family and/or carer support	118 (88.1)
Lives at a nursing home	3 (2.2)

**Table 2:** Clinical characteristics of the study patients.

Variable	n (%) T0 n = 134
Situation leading to the prescription of IBC:	
Post-surgical urinary bladder involvement	23 (17.2)
Impaired contractile function (no neurological disorder)	44 (32.8)
Neurogenic bladder	41 (30.6)
Neobladder	9 (6.7)
Bladder outlet obstruction (benign prostatic hyperplasia, prolapse)	4 (3.0)
Neurodegenerative disease (sclerosis)	10 (7.5)
Bladder–sphincter dyssynergia	3 (2.2)
Pre-existing conditions	
None	34 (25.4)
Cardiovascular conditions	33 (24.6)
Neurological conditions	25 (18.7)
Endocrine conditions	28 (20.9)
Respiratory conditions	8 (6.0)
Gastrointestinal conditions	11 (8.2)
Genitourinary conditions	27 (20.1)
Musculoskeletal conditions	26 (19.4)
Psychiatric conditions	7 (5.2)
Comorbidities	
None	77 (57.5)
Obesity	19 (14.2)
Prolapse	1 (0.7)
Benign prostatic hyperplasia	12 (9.0)
Muscle spasms	1 (0.7)
Previous depressions	13 (9.7)
Previous anxiety	13 (9.7)
Who indicated the IBC? (May include several)	
Nurse	15 (11.2)
Urologist	122 (91.0)
Gynaecologist	0 (0.0)
Physiatrist	8 (6.0)
Neurologist	0 (0.0)
Neurosurgeon	1 (0.7)
Number of catheterisations	
One	18 (13.4)
Two	37 (27.6)
Three	40 (29.9)
Four	28 (20.9)
Five	7 (5.2)
Six	3 (2.2)
Seven	1 (0.7)

**Table 3:** Characteristics relating to the ability to self-care.

Variable	n (%) T0
Hand function as reported by the patient	
Normal	109 (81.3)
Limited sensitivity, but with normal motor skills	15 (11.2)
Limited motor skills in the dominant hand	3 (2.2)
Limited motor skills in the NON-dominant hand	0 (0.0)
Limited motor skills in both hands	7 (5.2)
Mobility as reported by the patient	
Normal	81 (60.4)
Difficulty walking, but does not require help	20 (14.9)
Can walk with help	9 (6.7)
Uses a wheelchair, but could walk if needed	9 (6.7)
Permanently in a wheelchair	15 (11.2)
The patient has difficulty seeing the urinary meatus	
No	106 (79.1)
Yes	28 (20.9)
The patient can repeat the information on IBC provided by the nurse	
No	1 (0.7)
Yes	130 (97.0)
Unsure	3 (2.2)
The patient can follow the instructions given by the nurse	
No	4 (3.0)
Yes	124 (92.5)
Unsure	6 (4.5)
Who the patient thinks is going to perform the IBC	
I will (self-catheterisation)	118 (88.1)
With someone else’s help (assisted)	16 (11.9)

**Table 4:** Patients’ degree of concern over different problems that could be attributed to the catheterisation at the first visit.

Situations	Degree of Concern						
T0 n = 134	No Concern	A Little Concerned	Somewhat Concerned	Quite Concerned	Very Concerned	Md (IQR)	M (SD)
About inserting the catheter into their body	64 (47.8)	22 (16.4)	23 (18.2)	21 (15.7)	4 (3.0)	2. (2)	2.1 (1.24)
About getting an infection	35 (26.1)	23 (17.2)	42 (31.3)	27 (20.1)	7 (5.2)	3.0 (3)	2.6 (1.22)
About pain during catheterisation	63 (47.0)	23 (17.2)	27 (20.1)	15 (11.2)	6 (4.5)	2.0 (2)	2.1 (1.24)
About suffering an injury to the urethra	47 (35.1)	28 (20.9)	36 (26.9)	18 (13.4)	5 (2.7)	2.0 (2)	2.3 (1.19)
About loss of dignity	83 (61.9)	34 (25.4)	10 (7.5)	5 (3.7)	2 (1.5)	1.0 (1)	1.6 (0.90)
About loss of masculinity or femininity	96 (71.6)	29 (21.6)	7 (5.2)	2 (1.5)	0 (0.0)	1.0 (1)	1.4 (0.66)
About social rejection	94 (70.1)	26 (19.4)	8 (6.0)	4 (3.0)	2 (1.5)	1.0 (1)	1.5 (0.86)
About losing control of themself	75 (56.0)	33 (24.6)	21(15.7)	5 (3.7)	0 (0.0)	1.0 (1)	1.7 (0.87)

Md: Median; IQR: interquartile range; M: mean; SD: Standard deviation.

**Table 5:** Cognitive characteristics, quality of life, and psychological state of the study patients at the first visit (T0). HADS—Hospital Anxiety and Depression Scale.

Variable n = 134	Mean (SD)	n (%)
Cognitive state. Mini-Mental State Examination	31.7 (4.43)	
Normal (27 points or more)		122 (91.0)
Questionable (24–27 points)		5 (3.7)
Deterioration (12–24 points)		6 (4.5)
Dementia (<12 points)		1 (0.7)
Quality of life. King’s Health Questionnaire Dimensions		
General health perception	43.47 (19.85)	
Incontinence impact	56.7 (31.41)	
Role limitations	35.9 (31.32)	
Physical limitations	34.7 (35.46)	
Social limitations	26.4 (30.33)	
Personal relationships	58.7 (33.33)	
Emotions	26.5 (24.82)	
Sleep/energy	27.1 (32.16)	
Severity measures	33.6 (25.66)	
Psychological state. HADS		
Anxiety score	6.7 (4.11)	
Level of anxiety		
Normal		95 (70.9)
Borderline abnormal (borderline case)		23 (17.2)
Abnormal (case)		16 (11.9)
Depression score	4.78 (4.01)	
Level of depression		
Normal		111 (82.8)
Borderline abnormal (borderline case)		12 (9.0)
Abnormal (case)		11 (8.2)

**Table 6:** Characteristics of the catheter used and its use. Second visit.

Variable (T1)	n (%)
Type of catheter	n = 109
Hydrophilic catheter requiring internal activation or other pre-catheterisation step (break bag of built-in solution, unscrew connector, remove fluid from container, etc.)	13 (11.9)
Hydrophilic catheter requiring internal activation or other pre-catheterisation step (breaking bag of built-in solution, unscrew connector, remove fluid from container, etc.) with integrated diuresis bag	4 (3.7)
Catheter pre-lubricated with gel and with an integrated diuresis bag	6 (5.5)
Pre-lubricated hydrophilic ready-to-use catheter (with internal solution without activation required)	36 (33.0)
Pre-lubricated hydrophilic ready-to-use catheter (with internal solution without activation required) with integrated diuresis bag	5 (4.6)
Pre-lubricated hydrophilic ready-to-use catheter (with Vaporphilic Technology)	43 (39.4)
Pre-lubricated hydrophilic ready-to-use catheter with integrated diuresis bag (with Vaporphilic Technology)	2 (1.8)
Missing	3
Who performs the catheterisation	n = 112
The patient	95 (79.8)
Their partner	12 (10.1)
Another family member	4 (3.4)
External carer	1 (0.8)

**Table 7:** Evolution of adherence to and continued use of IBC. ICAS—Interpersonal Communication Assessment Scale.

Variable	T1 n = 104 n (%)	T2 n = 91 n (%)	T3 n = 88 n (%)	T1–T3 Comparison *p*-Value
Adherence classification according to the ICAS *				0.831
Strong	2 (1.9)	3 (3.3)	2 (2.3)	
Average	67 (64.4)	57 (62.6)	57 (64.8)	
Low	35 (33.7)	31 (34.1)	29 (33.0)	
ICAS adherence score [Mean (SD)] **	2.23 (1.42)	2.15 (1.51)	2.21 (1.46)	0.662
Maintains adherence (number of prescribed catheterisations) **	89 (84.8)	70 (77.8)	74 (84.1)	0.824

* McNemar’s test for paired data; ** Student–Fisher’s *t*-test for paired data.

**Table 8:** Difficulty performing bladder catheterisation at the three cut-off points.

	Frequency			T1–T3 Comparison	Intensity			T1–T3 Comparison
Situations	Mean T1 (SD) n = 104	Mean T2 (SD) n = 91	Mean T3 (SD) n = 88	*p*-Value	Mean T1 (SD) n = 104	Mean T2 (SD) n = 91	Mean T3 (SD) n = 88	*p*-Value
I’ve experienced pain	0.41 (0.58)	0.39 (0.65)	0.42 (0.63)	0.987	0.41 (0.57)	0.39 (0.67)	0.42 (0.63)	0.988
I’ve experienced bleeding	0.20 (0.43)	0.25 (0.46)	0.19 (0.39)	0.819	0.22 (0.48)	0.25 (0.46)	0.19 (0.39)	0.425
I can identify the meatus	0.39 (0.65)	0.23 (0.55)	0.14 (0.34)	0.009	0.39 (0.64)	0.21 (0.54)	0.14 (0.34)	0.009
I can open the catheter container	0.06 (0.27)	0.02 (0.14)	0.03 (0.18)	0.366	0.06 (0.27)	0.06 (0.28)	0.03 (0.18)	0.366
Activation/preparation of the catheter	0.06 (0.28)	0.06 (0.28)	0.02 (0.14)	0.206	0.06 (0.28)	0.04 (0.24)	0.02 (0.14)	0.206
Conduct self-catheterisation with “no touch” technique (prevent risk of bacterial contamination)	0.29 (0.50)	0.29 (0.54)	0.17 (0.38)	0.033	0.29 (0.52)	0.27 (0.53)	0.17 (0.38)	0.022
Conduct self-catheterisation (hardness or flexibility)	0.15 (0.40)	0.08 (0.31)	0.15 (0.38)	0.670	0.14 (0.40)	0.07 (0.30)	0.14 (0.34)	0.827
During catheterisation (insertion, progress, and removal)	0.51 (0.62)	0.44 (0.73)	0.33 (0.57)	0.092	0.51 (0.68)	0.44 (0.73)	0.33 (0.57)	0.112
Conduct self-catheterisation at social gatherings due to fear of spilling the container liquid onto myself	0.25 (0.58)	0.18 (0.46)	0.23 (0.47)	1.000	0.24 (0.56)	0.18 (0.46)	0.23 (0.47)	1.000
The container’s lack of discreetness causes me to avoid catheterisation when I am with other people	0.17 (0.47)	0.15 (0.41)	0.18 (0.53)	0.499	0.19 (0.54)	0.15 (0.41)	0.19 (0.53)	0.635
Public bathrooms do not meet hygienic requirements	0.94 (1.02)	0.88 (1.04)	1.15 (1.06)	0.165	0.93 (1.04)	0.89 (1.04)	1.15 (1.10)	0.147
Problems with accessing public bathrooms	0.49 (0.72)	0.56 (0.80)	0.82 (0.91)	<0.001	0.49 (0.72)	0.56 (0.80)	0.83 (0.90)	<0.001
I could not find a private place	0.78 (0.95)	0.77 (1.01)	1.02 (0.96)	0.023	0.77 (0.95)	0.76 (1.00)	1.02 (0.96)	0.016
I found it difficult to plan	0.48 (0.83)	0.36 (0.69)	0.45 (0.70)	0.692	0.47 (0.83)	0.35 (0.68)	0.44 (0.66)	0.613
Lack of help	0.07 (0.38)	0.09 (0.35)	0.10 (0.35)	0.058	0.06 (0.37)	0.08 (0.34)	0.08 (0.31)	0.058
Lack of time	0.17 (0.54)	0.08 (0.31)	0.18 (0.42)	0.670	0.17 (0.54)	0.08 (0.31)	0.17 (0.40)	0.670

Wilcoxon’s non-parametric test for paired data.

**Table 9:** Evolution of adherence, quality of life, and emotional state during follow-up between T1 and T6.

King’s Health Questionnaire Dimensions	Mean (SD) T0 n = 88	Mean (SD) T3 n = 88	Difference in Means	95% CI	*p*-Value
General health perception	42.78 (19.41)	38.40 (17.70)	4.38	−0.33; 9.09	0.068
Incontinence impact	57.39 (29.95)	40.55 (25.56)	16.84	8.95; 24.72	<0.001
Role limitations	36.42 (30.84)	20.10 (23.07)	16.32	9.01; 23.63	<0.001
Physical limitations	34.36 (34.93)	19.42 (23.28)	14.95	7.41; 22.48	<0.001
Social limitations	26.35 (29.48)	15.75 (20.97)	10.60	4.18; 17.02	0.001
Personal relationships	65.15 (31.14)	53.03 (27.71)	12.12	−12.96; 37.20	0.153
Emotions	24.63 (23.58)	16.04 (20.28)	8.59	2.48; 14.71	0.006
Sleep/Energy	27.66 (31.45)	10.48 (20.46)	17.18	10.18; 24.18	<0.001
Severity measures	32.85 (24.91)	26.39 (21.43)	6.46	0.95; 11.97	0.022
HADS					
Anxiety	6.62 (3.85)	6.02 (2.93)	0.60	−0.30; 1.50	0.190
Depression	4.53 (3.71)	4.74 (3.95)	−0.21	−1.20; 0.79	0.682

Student–Fisher’s *t*-test for paired data.

## Data Availability

The data that support the findings of this study are available from the author [BFLB], upon reasonable request.
